# Practice effects in a longitudinal, multi-center Alzheimer’s disease prevention clinical trial

**DOI:** 10.1186/1745-6215-13-217

**Published:** 2012-11-20

**Authors:** Erin L Abner, Brandon C Dennis, Melissa J Mathews, Marta S Mendiondo, Allison Caban-Holt, Richard J Kryscio, Frederick A Schmitt, John J Crowley

**Affiliations:** 1 Sanders-Brown Center on Aging, University of Kentucky, Lexington, KY, USA; 2 Departments of Neurology, University of Kentucky Lexington, Kentucky, USA; 3 Departments of Biostatistics, University of Kentucky, Lexington, KY, USA; 4 Departments of Statistics, University of Kentucky, Lexington, KY, USA; 5 Departments of Behavioral Science, University of Kentucky, Lexington, KY, USA; 6 Departments of Psychiatry, University of Kentucky, Lexington, KY, USA; 7 Cancer Research and Biostatistics, Seattle, WA, USA; 8 Sanders-Brown Center on Aging, University of Kentucky College of Medicine, Lexington, KY, USA

**Keywords:** Practice effects, Clinical trials, Alzheimer’s disease, Neuropsychological assessment

## Abstract

**Background:**

Practice effects are a known threat to reliability and validity in clinical trials. Few studies have investigated the potential influence of practice on repeated screening measures in longitudinal clinical trials with a focus on dementia prevention. The current study investigates whether practice effects exist on a screening measure commonly used in aging research, the Memory Impairment Screen (MIS).

**Methods:**

The PREADViSE trial is a clinical intervention study evaluating the efficacy of vitamin E and selenium for Alzheimer’s disease prevention. Participants are screened annually for incident dementia with the MIS. Participants with baseline and three consecutive follow-ups who made less than a perfect score at one or more assessments were included in the current analyses (N=1,803). An additional subset of participants with four consecutive assessments but who received the same version of the MIS at baseline and first follow-up (N=301) was also assessed to determine the effects of alternate forms on mitigating practice. We hypothesized that despite efforts to mitigate practice effects with alternate versions, MIS scores would improve with repeated screening. Linear mixed models were used to estimate mean MIS scores over time.

**Results:**

Among men with four visits and alternating MIS versions, although there is little evidence of a significant practice effect at the first follow-up, mean scores clearly improve at the second and third follow-ups for all but the oldest participants. Unlike those who received alternate versions, men given the same version at first follow-up show significant practice effects.

**Conclusion:**

While increases in the overall means were small, they represent a significant number of men whose scores improved with repeated testing. Such improvements could bias case ascertainment if not taken into account.

## Background

Serial cognitive assessment is used in clinical practice, clinical trials, and longitudinal studies of aging and dementia to track cognitive fluctuations over time and to identify clinically significant declines in performance suggestive of mild cognitive impairment (MCI) or dementia. Screening measures with specific cut-points reflecting probable cognitive impairment are also frequently used as brief, first-line measures of gross cognitive functioning in both clinical and research settings. For example, patients performing below the cut-point on a screening measure may be referred for more extensive diagnostic evaluation. Research participants may be screened into or out of studies based upon whether their performance lies above or below the cut-point of the measure. When cognitive instruments are used repeatedly, it is imperative to know not only the sensitivity, specificity, positive predictive value, and negative predictive value of the instruments, but also their behavior over time.

Practice effects (PE) represent one aspect of that behavior. PE are distinct from random fluctuations in performance and refer to bias due to familiarity with test items and procedures when a test is retaken [1]. Longitudinal studies of cognitive aging are highly dependent on repeated testing with neuropsychological measures. For example, dementia prevention trials such as the Alzheimer’s Disease Anti-inflammatory Prevention Trial (ADAPT) [2], Gingko Evaluation of Memory Study (GEMS) [3], and the Prevention of Alzheimer’s Disease by Vitamin E and Selenium (PREADViSE) trial [4] rely heavily on repeated cognitive screening measures and standardized cognitive batteries for case ascertainment and tracking response to treatment.

Most studies demonstrating practice effects have involved test-retest paradigms over short time intervals [5-9] or have been conducted primarily with impaired populations [10-12]. Nonetheless, repeated testing effects have been well documented [13-17], and performance variability has been demonstrated to be influenced by age [18-21], fluid intelligence [21], clinical population [10,22], retest interval [9,12,23,24], and the test or neurocognitive domain assessed [25,26]. Knowledge of the effects of repeated presentation is essential for interpretation of results. For example, PE can potentially alter the measure’s sensitivity to cognitive change and have been found to account for between 31 and 83% of the variance in follow-up test scores [26]. Further, PE could influence dementia detection in prevention trials when screening measures are used, especially given known PE, even for participants with Alzheimer’s disease (AD), on measures such as the Mini-Mental State Exam [12].

Furthermore, PE may persist over long periods of time. In the UK, Rabbit and colleagues [20] examined PE over a 17-year period in 5,899 participants, ages 49 to 92. Similar to other studies, they found the greatest gain in performance between the first and second presentation but observed gains due to practice on intelligence tests over intervals of several years. In a separate sample studied over a 20-year period, the same authors again observed significant PE, even with time intervals of up to four years [21]. Given this finding, it is also likely that PE may affect whether one performs above or below a single cut-point and thus influence case ascertainment in longitudinal clinical trials.

In the present study, we sought to examine PE on the Memory Impairment Screen (MIS) over four annual administrations. Brief memory screening instruments are often used in clinical practice and research to identify those patients who might benefit from a more extensive clinical assessment, and whether specific individuals should be included in a research study. Some studies, such as the PREADViSE trial, rely on dementia screening measures to determine whether a participant should be evaluated with more in-depth cognitive assessment. More specifically, if performance on screening measures is influenced by PE, participants who may be cognitively impaired or demented will be adjudicated as cognitively normal and thus misclassified or potentially lost to follow-up. Given previous data on short-term and long-term PE, we hypothesized that despite efforts to mitigate PE through alternate test versions, MIS scores would improve over time.

## Methods

### Participants

For details on recruitment and design of the National Institutes of Health (NIH) National Institute on Aging-sponsored PREADViSE trial, please see Kryscio *et al*. [4]. Briefly, the primary aim is to determine the effectiveness of the antioxidant supplements vitamin E and selenium in preventing the onset of AD. The PREADViSE trial recruited a subsample (n = 7,547) of participants age 62 and over (age 60 if of African-American descent) from the NIH National Cancer Institute-sponsored Selenium and Vitamin E Cancer Prevention Trial (SELECT) from 130 participating clinical sites in the US, Canada, and Puerto Rico. Men enrolled in both the SELECT prostate cancer study [27] and the PREADViSE trial, who completed baseline and three consecutive follow-up assessments, and obtained less than a perfect score at one or more assessments (n = 1,803) were included in the current analyses. Men with four consecutive assessments were selected to provide adequate follow-up to examine potential PE, and men with perfect scores at all assessments were excluded because their scores could not improve. However, these men (n = 1,291) were included in a sensitivity analysis.

Despite bi-annual training sessions on the screening protocol with the site clinical research assistants (CRAs) [28], there were administration errors that resulted in some men receiving the same MIS version at consecutive visits. Thus, an additional subset of men who received the same version of the test protocol (due to CRA error) at baseline and first follow-up and obtained less than a perfect score at any of the four assessments (n = 301) were also analyzed to determine the effectiveness of alternate forms in mitigating PE.

This study was approved by the University of Kentucky Institutional Review Board as well as the Institutional Review Boards at all participating centers.

### Clinical evaluation

The study employs a two-tier cognitive screening procedure for identification of memory impairment and dementia. The first consists of the MIS [29], which is administered at each annual visit. Participants who score below the predetermined cut-point on the MIS undergo a more extensive cognitive evaluation and medical work-up [28]. The MIS was chosen for its brevity (under five minutes) and ease of administration with minimal training by CRAs, who were well-versed in cancer research but had no other training or experience in administering cognitive tests. To minimize PE, the alternate form of the MIS [29] was also included in the protocol for subsequent annual assessments. At each follow-up screen, the participant received the version not administered to him the previous year. During MIS administration, the participant is shown four written words and verbally given a category cue for each; after a 2-minute interval filled with a non-memory-based distraction task, the participant is asked to recall the words (free recall). Category cues may be given as needed to stimulate recall (cued recall). Two points are awarded for each correct free recall word, and one point is scored for each correct word following category cue. MIS total scores range from 0 to 8 points with 8 points indicating a perfect score, and a standard cut-point, recommended by Buschke and the test authors [29] is a score of 4. MIS screening began in May 2002 and will continue through January 2013. The cut-point was raised to 5 in January 2009 to capture participants potentially functioning in the MCI range.

### Statistical methods

Linear mixed models (LMM) were used to test the hypothesis that MIS scores improve over time due to PE. Random intercepts and an unstructured covariance matrix were used to account for within-subject correlation. Initial models included fixed effects for age at baseline (centered at 70), education level (high school or lower, college or higher), race (African-American vs. not African-American), MIS version (version 1 vs. version 2), and annual visit, which was treated as acategorical variable. Two-way interactions between visit and age, race, and education were then added to the model.

Standard two-group comparisons (for example, *t*-tests and chi-square (*χ*^2^) tests) were used to assess comparability between the men who received alternating versions over four visits and those who received the same version at baseline and first follow-up. Statistical significance was set at α = 0.05. All analyses were performed with SAS/STAT 9.3® software.

## Results

Participants had an average ± SD baseline age of 68.0 ± 5.3 years and were highly educated, with 75.6% percent reporting at least some college education (Table 1). Participants with alternating MIS versions had received similar levels of education to those given the same version at baseline and follow-up but were slightly younger (*t* = −2.18, degrees of freedom (df) = 380.88, *P* = 0.03) and comprised fewer minorities (*χ*^2^ = 33.4, df = 2, *P* < 0.0001). Almost half the men (47.0%) who received alternating versions obtained a perfect score at baseline (Table 2), and about half (49.8%) of those maintained their perfect score at the first follow-up screen (data not shown). Among men who did not achieve a perfect baseline score (n = 955), 70.5% improved their score at the first follow-up screen while just 6.0% performed worse. Between baseline and follow-up visit 3, a 16.2% increase in the proportion of men obtaining a perfect score was observed along with corresponding decreases in the proportion of men obtaining less than perfect scores (McNemar’s *χ*^2^ = 5.5, df = 1, *P* = 0.02) (Table 2). By contrast, although the same proportion of men (47.0%) made a perfect score at baseline, a 14.0% increase in perfect scores was observed between baseline and follow-up visit 1 among the men who received the same version at the two assessments (McNemar’s *χ*^2^ = 10.9, df = 1, *P* = 0.001) (data not shown).

**Table 1:** Baseline participant characteristics

**Characteristics**	**Alternating versions**	**Same version**
	**(n = 1,803)**	**(n = 301)**
**Age, years, mean (SD)**	67.8 (5.2)	68.6 (5.9)
**Race, n (%)**		
African-American	133 (7.4)	27 (9.0)
White	1629 (90.4)	249 (82.7)
Other	41 (2.3)	25 (8.3)
**Education level, n (%)**		
Unknown	8 (0.4)	0 (0.0)
High school or lower	447 (24.8)	63 (20.9)
Some college or higher	1348 (74.8)	238 (79.1)

**Table 2:** Memory Impairment Screen (MIS) scores by visit

**All participants with alternating versions (n = 1,803)**				
	**Baseline MIS**	**FU 1 MIS**	**FU 2 MIS**	**FU 3 MIS**
MIS score, mean (SD)	7.34 (0.74)	7.47 (0.68)	7.48 (0.70)	7.54 (0.69)
MIS score, n (%)				
≤ 5	45 (2.5%)	24 (1.3%)	30 (1.7%)	33 (1.8%)
6	151 (8.4%)	123 (6.8%)	128 (7.1%)	103 (5.7%)
7	759 (42.1%)	632 (35.1%)	596 (33.1%)	527 (29.2%)
8	848 (47.0%)	1,024 (56.8%)	1,049 (58.2%)	1,140 (63.2%)
Total	1,803 (100%)	1,803 (100%)	1,803 (100%)	1,803 (100%)
**Participants age 75 years or more at baseline with alternating versions (n = 218)**				
	**Baseline MIS**	**FU 1 MIS**	**FU 2 MIS**	**FU 3 MIS**
MIS score, mean (SD)	7.30 (0.81)	7.41 (0.71)	7.33 (0.76)	7.32 (0.82)
MIS score, n (%)				
≤ 5	8 (3.7%)	2 (0.9%)	5 (2.3%)	10 (4.6%)
6	24 (11.0%)	22 (10.1%)	23 (10.6%)	19 (8.7%)
7	80 (36.7%)	79 (36.2%)	84 (38.5%)	80 (36.7%)
8	106 (48.6%)	115 (52.8%)	106 (48.6%)	109 (50.0%)
Total	218 (100%)	218 (100%)	218 (100%)	218 (100%)

FU, follow-up visit.

For the men with four visits and alternating MIS versions, all main effects except race were significant in the initial LMM. Two-way interactions of age, education, and version with visit were significant when added to this model. A three-way interaction among age, education, and visit was also tested and was not significant. Hence, the final LMM contained random intercepts (that is, subject effects); main effects for age, education, visit, and MIS version; and visit by age, visit by education, and visit by version interaction terms. The effects of age and education in this model are illustrated in Table 3. While there were few significant PE at the first follow-up, which is unremarkable given almost half the men already had a perfect score at baseline, the youngest men (age 60 years) and men with the highest educational level did show a significant increase over baseline and maintained this PE through the third follow-up. By the second follow-up assessment a significant increase over baseline, which was maintained at the third follow-up, was observed for both educational levels and for all men age 70 years or younger. Although their mean scores did not decrease monotonically, men age 75 years and older showed few significant PE and tended to have lower estimated scores over time. Only the estimated mean scores at follow-up visit 2 for men age 75 years were significantly higher than baseline, while by follow-up visit 3 men age 85 years had estimated mean scores significantly lower than baseline. Finally, between baseline and follow-up visit 3, the proportion of men age 75 years and older at baseline (n = 218) who achieved a perfect score increased by just 1.4% in contrast to 70.5% for the entire sample receiving alternate forms (Table 2).

**Table 3:** Adjusted mean Memory Impairment Screen (MIS) scores based on a linear mixed model (LMM): alternating versions from baseline through follow-up (FU) visit 3 (n = 1,803)

**LMM factor**	**Baseline MIS**	**FU 1 MIS**	**FU 2 MIS**	**FU 3 MIS**
Education				
≤ High school	7.29 (0.03)	7.28 (0.03)	7.45 (0.03)^1,2^	7.53 (0.03)^1,2^
≥ Some college	7.40 (0.02)	7.46 (0.02)^1^	7.53 (0.02)^1,2^	7.53 (0.02)^1,3^
Age, years				
60	7.37 (0.03)	7.46 (0.03)^1^	7.61 (0.03)^1,2^	7.71 (0.03)^1,2,3^
65	7.36 (0.02)	7.41 (0.02)	7.53 (0.02)^1,2^	7.59 (0.02)^1,2^
70	7.34 (0.02)	7.35 (0.02)	7.46 (0.02)^1,2^	7.48 (0.02)^1,2^
75	7.33 (0.03)	7.29 (0.03)	7.38 (0.03)^2^	7.36 (0.03)
80	7.31 (0.04)	7.23 (0.04)	7.31 (0.04)	7.25 (0.04)
85	7.29 (0.06)	7.17 (0.06)	7.23 (0.06)	7.13 (0.06)^4^

Results are presented as mean score (SEM).^1^significantly higher than baseline; ^2^significantly higher than FU 1; ^3^significantly higher than FU 2; ^4^significantly lower than baseline.

Results changed little when men who obtained a perfect score at all four assessments were included in the analysis. While there were no significant PE at follow-up visit 1 for any age or educational level, PE were first observed among the youngest and best educated participants, while the oldest participants (age 80 years or more) had significantly lower estimated mean MIS scores at the third follow-up than at baseline.

For the men who were given the same MIS version at baseline and first follow-up (Table 4), only main effects for age, education, version, and visit were significant predictors of MIS score. For these men, there was a significant improvement from baseline in the overall mean at the first follow-up (0.20 ± 0.05 point increase, *P* = 0.0005), and this holds true for even the oldest participants (0.17 ± 0.07 point increase, *P* = 0.02). Further, at follow-up visit 2, where 75% of participants received the alternate version for the first time, there was a non-significant decrease in the overall mean (0.10 ± 0.06 points, *P* = 0.098), all of which underscores the importance of alternating instrument versions to minimize PE in screening. These men were quite similar to those who received alternating versions of the MIS on all baseline characteristics except race, which is likely due to MIS administration errors occurring more often at certain study sites that happened to have more non-white participants. Since race was not a significant predictor in this analysis, however, the higher proportion of African-Americans and other races in this group does not explain the PE.

**Table 4:** Adjusted mean Memory Impairment Screen (MIS) scores based on a linear mixed model (LMM): same version at baseline and follow-up (FU) visit 1 (n = 301)

**LMM Factor**	**Baseline MIS**	**FU 1 MIS**	**FU 2 MIS**	**FU 3 MIS**
Education				
≤ High school	7.25 (0.05)	7.36 (0.09)	7.31 (0.09)	7.40 (0.10)
≥ Some college	7.35 (0.05)	7.57 (0.05)^1^	7.45 (0.05)	7.65 (0.05)^1,2^
Age, years				
60	7.48 (0.07)	7.65 (0.07)^1^	7.57 (0.07)	7.71 (0.07)^1^
65	7.38 (0.06)	7.54 (0.06)^1^	7.46 (0.06)	7.60 (0.06)
70	7.27 (0.05)	7.43 (0.05)^1^	7.35 (0.06)	7.49 (0.05)^1^
75	7.16 (0.06)	7.32 (0.06)^1^	7.24 (0.06)	7.39 (0.06)^1^
80	7.05 (0.07)	7.22 (0.07)^1^	7.14 (0.07)	7.28 (0.07)^1^
85	6.94 (0.09)	7.11 (0.09)^1^	7.03 (0.09)	7.17 (0.09)^1^

Results are presented as mean score (SEM).^1^significantly higher than baseline; ^2^significantly higher than FU 2.

## Discussion

Determining the success or failure of a dementia prevention trial depends heavily on the ability of the investigators to ascertain caseness. In a large trial, where budget and time constraints may dictate the use of uncomplicated screening instruments, unrecognized PE may mask impairment and consequently bias results. In such cases it is desirable to minimize PE to identify individuals who need further evaluation.

We examined PE over four annual presentations of a brief memory screen, the MIS. In contrast to several previous studies of other instruments, we found a robust PE between the first and second presentation only when identical test forms were used. Use of alternate versions largely mitigated the PE at first follow-up, although PE was observed for those with at least some college education and for the youngest participants, which is consistent with findings from other studies. Interestingly, there were few PE for the oldest participants when alternate forms were used consistently. In fact, similar to the findings from the 5-year *Personnes Agées QUID* (PAQUID) study [30], these participants tended to do worse over time, which may support the hypothesis that a lack of PE may signal early cognitive decline [8,31,32].

The study population consisted only of men, and therefore potential gender differences could not be studied, therefore the generalizability of findings to women is uncertain. Moreover, treatment effects of vitamin E and selenium, if they exist, could not be assessed as the investigators remain blinded to treatment arm. The utility of these results is limited by the nature of the MIS, an exclusively memory-based measure that neglects other areas of cognitive functioning. In addition, because of the restricted range and clear ceiling effect with this instrument, men who had a perfect score could not improve; the floor effect was not a factor since none of the participants in our study scored zero. However, the relatively small mean increases in scores between visits reflect the limited range of the MIS and should not be mistaken for clinically insignificant changes. It is notable that PE of any magnitude were found on a brief, four-item screening measure with identical versions being presented two years apart. Further, while the changes in the means were small, the proportion of perfect scores increased steadily and quite dramatically over time, if alternating versions were not used. These results continue to support the use of alternate forms in clinical and research settings where identifying candidates for further evaluation is the goal.

This study contributes to the literature in several ways. First, it adds to the information on the variability of cognitive screening measures across long periods of time, especially for longitudinal aging trials. It also adds to the information on the performance of the MIS as a brief screening measure for participants of varying age, education, and ethnic background. These data should further serve to inform the design and implementation of future dementia prevention studies.

Although there are several longitudinal studies investigating reliable change indices (RCI), we view this as an issue that is related to but separate from PE. More specifically, RCI allow one to control for the effect of practice in determining whether there has been a reliable change in cognition over time. Screening measures used in longitudinal studies are typically not used to detect subtle declines per se but rather to re-screen participants for inclusion into or exclusion from a study. Additionally, some studies have shown that RCIs must be rather large to reflect credible change [33-37]. However, a PE of just one point can be consequential enough to have detrimental effects on case ascertainment.

## Conclusion

In this paper, we present the results of PE on a sample of 2,104 cognitively intact adult men over age 60 years, tested annually over four years. Strengths of the design itself include the large sample size, longitudinal nature of the study, and use of alternate forms for the vast majority of examinees. This study also demonstrates subtle but important shifts toward improved scores over time on a brief screening measure. Given the importance of repeated brief screening measures to clinical trial case ascertainment, our study highlights the importance of evaluating the effect of practice on specific instruments used in longitudinal clinical trials. Future research may wish to explore the possibility of adjusting cut-points on repeated measures, and determining the effect this might have on overall case ascertainment.

## Competing interests

The authors declare that they have no competing interests.

## Authors’ contributions

ELA prepared the data, performed all statistical analyses, helped draft the manuscript, and revised this manuscript for resubmission to *Trials*. BCD helped draft the manuscript. MJM helped draft and revise the manuscript. MSM participated in the design of the study and helped draft the manuscript. ACH trained site staff on the screening protocol and participated in study coordination. RJK participated in the design of the study, oversaw the statistical analysis plan, and helped draft the manuscript. FAS participated in the design and coordination of the study, trained site staff on the screening protocol, and helped draft the manuscript. JCJ participated in the design of the study and helped coordinate data collection. All authors read and approved the final manuscript.
